# Population-based utility scores for HPV infection and oropharyngeal squamous cell carcinoma among Indigenous Australians

**DOI:** 10.1186/s12889-021-11496-z

**Published:** 2021-07-26

**Authors:** Xiangqun Ju, Karen Canfell, Kirsten Howard, Gail Garvey, Joanne Hedges, Megan Smith, Lisa Jamieson

**Affiliations:** 1 Australian Research Centre for Population Oral Health, Adelaide Dental School, The University of Adelaide, Adelaide Health & Medical Sciences Building, Adelaide, 5005 Australia; 2 Cancer Council of NSW, Sydney, Australia; 3 School of Public Health, The University of Sydney, Sydney, Australia; 4 Menzies School of Health Research, Darwin, Australia

**Keywords:** Utility scores, Oropharyngeal squamous cell carcinoma (OPSCC), Human papillomaviruses (HPV), Vaccination, Indigenous Australians

## Abstract

**Background:**

Oropharyngeal squamous cell carcinoma (OPSCC) is associated with high mortality. Human papillomavirus (HPV) infection is a significant risk factor for OPSCC. Utilities are fundamental values representing the strength of individuals’ preferences for specific health-related outcomes. Our study aim was to work in partnership with Indigenous communities in South Australia to develop, pilot test and estimate utility scores for health states related to HPV, HPV vaccination, precursor OPSCC and its treatment, and early stage OPSCC among Indigenous Australians.

**Methods:**

Development and pilot testing of hypothetical HPV and OPSCC health states, specifically through the lens of being Indigenous Australian, was conducted with an Indigenous Reference Group. Six health states were decided upon, with utility scores calculated using a two-stage standard gamble approach among a large convenience sample of Indigenous Australians aged 18+ years residing in South Australia. The rank, percentage of perfect health and utility score of each health state was summarised using means, and medians at 12 months and lifetime duration. Potential differences by age, sex and residential location were assessed using the Wilcox Rank Sum test.

**Results:**

Data from 1011 participants was obtained. The mean utility scores decreased with increasing severity of health states, ranging from 0.91–0.92 in ‘screened, cytology normal, HPV vaccination’ and ‘screened, HPV positive, endoscopy normal’, to less than 0.90 (ranging from 0.87–0.88) in lower grade conditions (oral warts and oral intraepithelial neoplasia) and less than 0.80 (ranging from 0.75–0.79) in ‘early stage throat cancer’. Higher utility scores were observed for ‘screened, cytology normal and HPV vaccination’ among younger participants (18–40 years), for ‘early stage invasive throat cancer’ among females, and for ‘oral intraepithelial neoplasia’ and ‘early stage invasive throat cancer’ among metropolitan-dwelling participants.

**Conclusion:**

Among a large sample of Indigenous Australians, utility for oral HPV infection and OPSCC decreased with severity of health states. Older participants, as well as males and those residing in non-metropolitan locations, had decreased utility for high-grade cytology and early invasive cancer states. Our findings are an important contribution to cost-utility and disease prevention strategies that seek to inform policies around reducing HPV infection and OPSCC among all Australians.

**Supplementary Information:**

The online version contains supplementary material available at 10.1186/s12889-021-11496-z.

## Background

Oropharyngeal squamous cell carcinoma (OPSCC), which occurs in the middle part of the throat, including the tonsils, posterior one-third of the tongue, and lateral and posterior walls of the oropharynx [1], is a serious condition associated with high mortality. Advanced OPSCC affects eating, swallowing, speaking and leads to very poor quality of life. The incidence of OPSCC has increased over the last 20 years in several developed countries [2], including Australia [3], Denmark [4], the United States [5] and the United Kingdom [6]. Survival from OPSCC is comparatively low because it is frequently asymptomatic and therefore diagnosed at a late stage. Relative 5-year survival rate in 2011–2015 in Australia was 68.6% [7], and 70% in the United States [8]. Indigenous Australians (Australians who identify as being of Aboriginal and/or Torres Strait Islander descent) have a higher incidence ratio (2.16) of OPSCC [9, 10], and lower five-year survival (43%) than non-Indigenous Australians (75%) [11].

In addition to tobacco use and alcohol consumption, human papilloma virus (HPV) 16 infection has been increasingly identified as a significant risk factor for OPSCC [12]. Among non-smoking young adults, the risk of HPV16 infection is increased three- to five-fold who have experienced a number of sexual partners compared to those with fewer sexual partners [13]. The proportion of OPSCC that is attributable to HPV16 has increased over the last decade in Europe and North America, to an estimated 70% [14]. Indicative data from Australia suggests a similar increase in the fraction of OPSCCs that might be attributable to HPV infection [15]. However, there are currently no population estimates of HPV-related OPSCC among Indigenous Australians.

Prevention of infection with the most aggressive oncogenic HPV types through HPV vaccination has been in place in Australia since 2007 and is expected to decrease the burden of HPV-related cancers over the longer term. Population-level impacts and herd effects following HPV vaccination have been documented [16], specifically among Indigenous Australians [17–19]. While coverage is similar in Indigenous and non-Indigenous adolescents for the first dose of the vaccine course, course completion rates are generally lower in Indigenous adolescents [20]. In light of the higher burden of HPV-related cancers (e.g. OPSCC and cervical cancer) among Indigenous Australians, catch-up vaccinations for those who did not receive a full vaccine course may be warranted. Evaluation of these strategies requires health state utilities in relation to HPV-related disease for Indigenous Australians, which do not currently exist. Health state utilities for oral HPV infection and OPSCC are rare for non-Indigenous populations and non-existent for Indigenous Australians [16, 21, 22].

Utilities are numbers that represent the strength of an individual’s preference for specific health states, measured on a dead (0) to full health (1) scale. Indigenous peoples’ own values and preferences for health states need to be prioritised, as there should be no assumption that these are the same as those of non-Indigenous people [23]. The aim of this study was to work in partnership with Indigenous communities in South Australia to develop, pilot test and estimate utility values for health states related to HPV infection, HPV vaccination, precursor OPSCC and its treatment, and early stage OPSCC by age, gender and location groups among Indigenous Australians.

## Methods

### Development of Health states

An Indigenous Reference Group (IRG) comprising several respected Indigenous adults with diverse backgrounds from across South Australia was convened to develop and test the health state descriptions used to estimate utilities through an Indigenous lens. The IRG included Indigenous community members, councillors and health workers, and was chaired by an Indigenous health manager. Domains that the IRG recognised as being of fundamental importance in the health state preferences included: (1) racism/distrust/confusion of health sector (with anticipation of racism being very strong); (2) connection/responsibilities to family; (3) social determinants of health uniquely over-represented in many Indigenous families (death, incarceration, child removal from family by state, poverty, domestic violence, addictions, food insecurity, loss and grief, hum-bugging (concept of ‘what’s yours is mine’); (4) connections with country (especially for remote-dwelling participants) and; (5) spiritual thought processes; acceptance that sickness is their lot, accepting cancer is being ‘sung to death’; going to the ancestors. An initial 10 health states were developed, which included: (1) cytology normal; (2) HPV vaccination (3) low-grade oral cytology; (4) low-grade cytology with endoscopy; (5) HPV positive and cytology normal; (6) HPV positive and endoscopy normal; (7) oral warts; (8) high grade cytology with histologically-confirmed Grade I oral intraepithelial neoplasia; (9) high grade cytology with histologically-confirmed Grade II/III oral intraepithelial neoplasia and; (10) invasive squamous cell oropharyngeal carcinoma. However, in the pilot testing phase (*n* = 8 Indigenous adults not included in the main study), it became apparent that the participant burden from 10 health states was too great. In response, four health states (#3, #4, #5 and #8) were deleted, with the remaining six included in the final questionnaire. The IRG considered it imperative to hire and train research officers who were able to respectfully engage and be responsive to the cultural values of participants, so that participants felt comfortable during the interview and that there was interviewer consistency. This was achieved by the IRG being actively involved in the recruitment and training of research officers.

### Data collection

Health state data was obtained from a large convenience sample of Indigenous Australians aged 18+ years residing in South Australia as part of a broader study examining oral HPV infection and OPSCC [24]. Briefly, participants were recruited through Aboriginal Community Controlled Health Organisations (ACCHOs), who were key stakeholders in the study. All participants provided signed informed consent. Data was collected from February 2018 to January 2019.

### Health state scenarios

The six hypothetical HPV-related OPSCC health states developed and piloted by the IRG (Table 1) were evaluated in the larger convenience sample via face-to-face interview by four trained research officers. The scenario descriptions (Additional file 1) were informed by relevant Australian cancer screening and treatment guidelines, including the psychosocial literature in relation to Indigenous health, and incorporated the feedback from the IRG [18, 25–27]. Each scenario was described in a narrative format with the use of visual prompts and aids. Participants were invited to ask as many questions as they liked for clarification purposes. Participants were then asked to rank the description of each health state relative to the others, from one to six (one being most desirable, six being least desirable; equal ranking was accepted). At no stage were participants asked if they, or anyone they knew, had experienced any of the health states. The participant interviews averaged 1 h in duration (ranging from 45 min to 1 h 20 min). As with any population group unfamiliar with standard gamble procedures, this time was critical to ensure participants understood the scenarios and that the values provided were both meaningful and an accurate portrayal of how participants viewed and framed the health states.

**Table 1:** Hypothetical health state scenarios relating to oral HPV infection and oropharyngeal cancer, ranks and percentages of perfect health by six health states lasting 12 months, standard gamble utility scores and intra-class correlation coefficient for OPSCC duration among Indigenous adults (*n* = 1011)

Codes	Health states	Description	Rank	Perfect health lasting 12 months (%)		Oropharyngeal cancer duration				
						12 months		Lifetime		
			Median (IQR)	Mean (SD)	Median (IQR)	Mean (SD)	Median (IQR)	Mean (SD)	Median (IQR)	ICC
S1	Screened; cytology normal	OPSCC screening test cytology negative (Roger)	1 (1)	85.1 (16.7)	90.0 (20.0)	0.91 (0.28)	1.00 (0.04)	0.91 (0.28)	1.00 (0.10)	0.99959
S2	HPV vaccination	Three doses of HPV vaccine (Emily)	2 (2)	79.8 (19.2)	85.0 (23.5)	0.92 (0.27)	1.00 (0.01)	0.91 (0.28)	1.00 (0.01)	0.99793
S3	Screened; HPV positive, endoscopy normal	HPV positive and cytology negative; follow-up OPSCC screening in 12 months (Sam)	3 (1)	69.0 (18.2)	70.0 (20.0)	0.92 (0.28)	1.00 (0.01)	0.91 (0.28)	1.00 (0.01)	0.99309
S4	Oral warts	Treatment for oral warts associated with HPV infection (Rachel)	4 (1)	68.0 (20.5)	70.0 (26.5)	0.88 (0.32)	1.00 (0.00)	0.88 (0.32)	1.00 (0.00)	0.99998
S5	HG cytology with OIN II-III	High grade cytology with histologically-confirmed grade II/III oral intraepithelial neoplasia (Max)	5 (0)	48.6 (20.4)	50.0 (20.0)	0.87 (0.34)	1.00 (0.14)	0.87 (0.34)	1.00 (0.11)	0.99664
S6	Early stage invasive throat cancer	Early stage invasive throat cancer requiring laryngectomy (Mary)	6 (0)	25.9 (21.5)	20.0 (30.0)	0.75 (0.43)	1.00 (0.81)	0.79 (0.40)	1.00 (0.00)	0.45521

Interquartile range (IQR) being the difference between 75th (Q_3_) and 25th (Q_1_) percentiles (IQR = Q_3_-Q_1_)

*HPV* human papillomavirus, *OPSCC* oropharyngeal squamous cell carcinoma, *HG* high grade, *OIN* oral intraepithelial neoplasia

### Health state preference score assessment

**Fig. 1:**
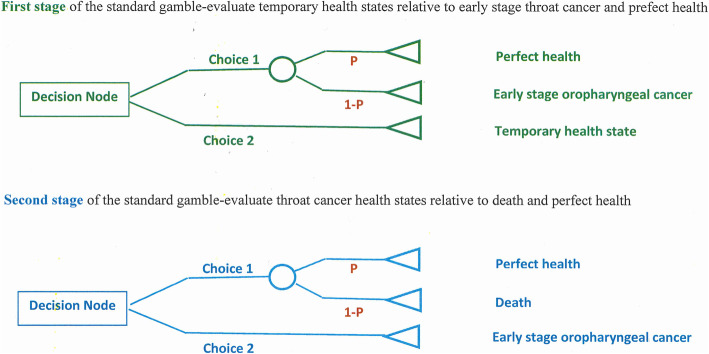
Decision tree of two-stage standard gamble utilities

Utility scores for each of the six hypothetical health states were assessed using a two-stage standard gamble approach (Fig. 1) [28]. As a method, the two-stage standard gamble aims to measure the ‘Utility’ of a health state by observing an individual’s willingness to accept the likelihood of death in order to avoid the postulated health state. For the five temporary health states (from non-cancer to early stage OPSCC), participants were asked to imagine health returning to back to how it initially was after 12 months (Stage 1: Green colour in Fig. 1). For instance, selecting ‘Choice 2’ means the chance (the probability = 100%) of living with the temporary health state for 12 months, followed by perfect health for the rest of your life; selecting ‘Choice 1’ means you are happy to take the gamble (with probability ranging from 50 to 99%) of perfect health or the chance of living with early stage OPSCC for 12 months, followed by perfect health for the rest of your life. For the early stage invasive throat cancer health state, where the probability of indifference between living with early stage throat cancer is measured relative to the risky prospects associated with either perfect health or immediate death (Stage 2: Blue colour in Fig. 1), two ‘time in state’ durations were used. The first was for 12 months followed by sudden and painless death, the second was from the present until age 85 years, followed by sudden and painless death. Participants therefore provided seven health state preference scores: five scores for five temporary health states and two scores for early stage invasive throat cancer at 12 months and lifetime.

Stage 1 derived utilities for each of the health states, measured on the scale of perfect health and early stage invasive OPSCC (termed ‘throat cancer’ after recommendation from the IRG). Stage 2 derived utilities for early stage invasive throat cancer (‘Mary’ vignette) on the scale of perfect health to death. Death was described as ‘being finished’, again following advice from the IRG. Details of the algorithms used to transform states from stage 1 to death (0) – full health (1) scale of a conventional utility are described in the analysis section below.

### Sample demographic data

In addition to the standard gamble exercise, data on participant age, sex and residential location were collected, and dichotomized ‘18–40 years’ and ‘> 40 years’, male’ and ‘female’ and ‘metropolitan’ and ‘non-metropolitan’, respectively.

### Statistical analysis

To determine utility scores for the temporary health states on a 0–1 cardinal interval scale, scores were mathematically transformed using the following function: *h*_*i*_ = *P*_*i*_ + (1 - *P*_*i*_) *h*_*k*_ where *h*_*i*_ is the utility of the temporary health state, *P*_*i*_ is the probability of indifference observed between the certain outcome of experiencing the temporary health state and the risky prospect of either living with early stage throat cancer or living with perfect health. *h*_*k*_ is the utility of early stage throat cancer (worst health outcome) evaluated on the death to perfect health scale [28]. For early stage throat cancer there are thus two *h*_*k*_; one evaluated on the 12-month time scale, the other evaluated on the life time scale. For each individual utility score representing a temporary health state we applied two separate ‘time in state’ values representing the anchor state (using the mathematical function *h*_*i*_ = *P*_*i*_ + (1 - *P*_*i*_)*h*_*k*_). This resulted in two distinct utility scores on the 0–1 cardinal interval scale for each participant’s temporary health state, characterised by the ‘time in state’ value; yielding 10 temporary health state utility scores for each participant.

Demographic characteristics were described by number and percentage. Six health states were ranked, with the percentage of perfect health at 12 months estimated. The utility score of each health state was summarised using means and standard deviation (SD) as well as medians and inter-quartile range (IQR). Intra-class correlation coefficients (ICC) were used to assess the level of agreement between the pair of ‘early stage throat cancer’ scores evaluated using ‘12 months’ and ‘lifetime’ durations (‘time in state’ values) [29]. ICC was evaluated with the following formula:

$$ ICC=\left({MS}_R-{MS}_E\right)/{MS}_R+\left(k- 1\right)\ {MS}_E+k/n\ \left({MS}_c+{MS}_E\right) $$

Where ***MS*** is mean square, ***MS***_***R***_ is characterised as the difference between the grand mean for a health state (combining both sets of utility scores calculated using different ‘time in state’ values) and the group means for a health state calculated according to a specific ‘time in state’ value. ***MS***_***c***_ is characterised as the difference between the specific ‘time in state’ individual utility scores and the mean of these scores. ***MS***_***E***_ is mean square for error, ***n*** is the number of participants and ***k*** is measurement value. ICC values above 0.90 were considered ‘excellent’, between 0.75–0.90 and 0.50–0.75 were ‘good’ and ‘moderate’ agreement respectively, while those less than 0.50 were considered ‘poor’ [30]. To enable calculation of ICCs, two-way mixed effects, absolute agreement and single measurement were carried out for each health state, such that mean square values (‘between groups’ and ‘within groups’) were determined for utility scores based on each ‘time in state’ anchor point [29, 30].

The Wilcoxon Rank Sum tests were used to assess differences in the distribution of utility scores for each temporary health state according to age, sex and residential location [31]. Utility scores transformed with ‘12 month’ and ‘lifetime’ durations were analysed separately using mean and 95% confidence interval (95% CI) for each demographic outcome. Statistically significant differences were denoted by non-over-lapping 95% confidence intervals. Given six distinct health states were being tested, with two sets of ‘time in state’ anchor states for the mathematical transformation of temporary health states, a total of 18 tests for statistical significance were made.

All variables had less than 3% missing values (ranging from 1.7% for health status ‘Screened; cytology normal’ at 12 months to 2.8% for health status ‘Early stage invasive throat cancer’ at lifetime), so an available data was used for data analysis. SAS statistical software (SAS 9.4, SAS Institute Inc., Cary, NC, USA) was used to analyse data.

## Results

A total of 1011 Indigenous Australians residing in South Australia aged 18+ years completed the health state utilities questionnaire. The average age was 39.8 (standard deviation =14.8, ranged from 18 to 82) years, with 46.6% aged 40 years or older. Two-thirds (66.4%) were female and 62.7% resided in non-metropolitan locations. The ordinal rank of each health state, and percentage of perfect health on each health state lasting 12 months, is presented in Table 1. The highest ranked was ‘screened, cytology normal’ (median rank: 1; IQR: 1–2), with an average of 85% of perfect health lasting 12 months. The lowest ranked was ‘early stage invasive throat cancer’.

**Fig. 2:**
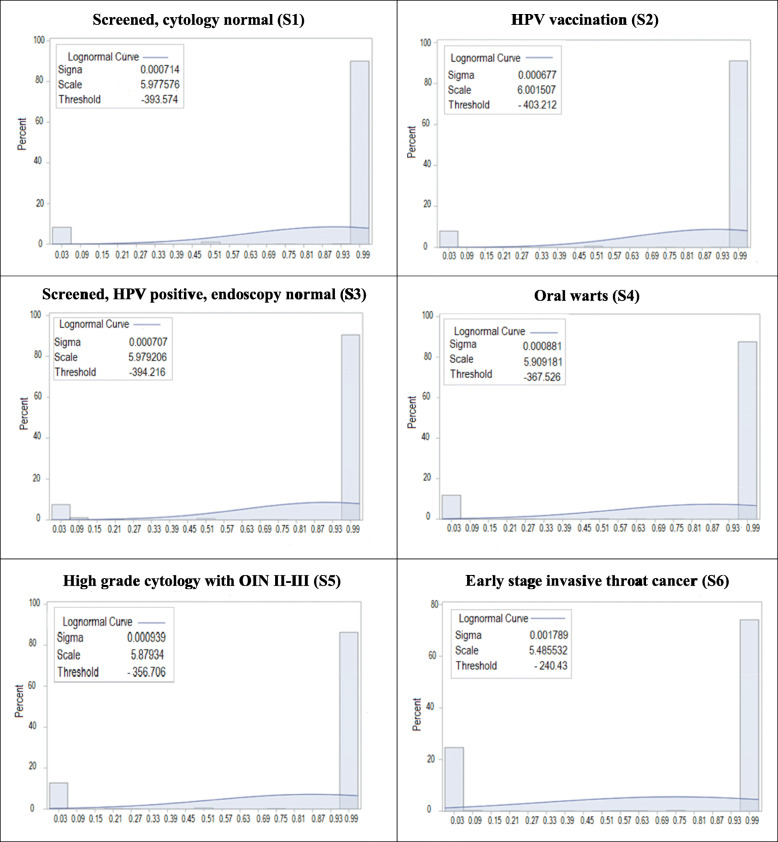
Standard gamble utility score distributions at the ‘12 month’ duration early stage throat cancer. S1: screened, cytology normal. S2: HPV vaccination. S3: screened; HPV positive, endoscopy normal. S4: oral warts. S5: high grade cytology with OIN II-III. S6: early stage invasive throat cancer

**Fig. 3:**
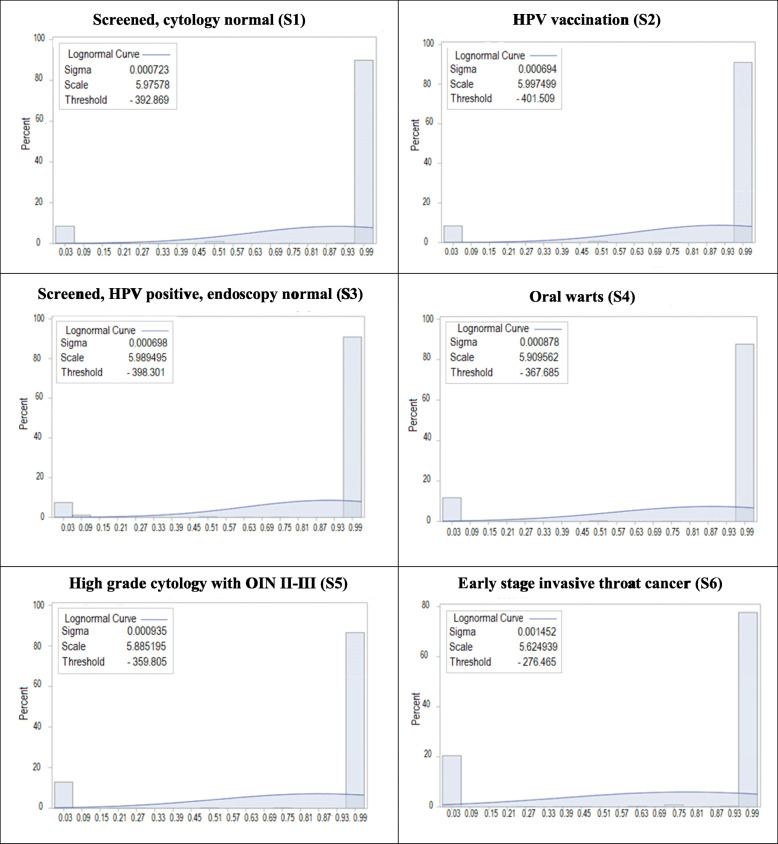
Standard gamble utility score distributions at ‘lifetime’ duration early stage throat cancer. S1: screened, cytology normal. S2: HPV vaccination. S3: screened; HPV positive, endoscopy normal. S4: oral warts. S5: high grade cytology with OIN II-III. S6: early stage invasive throat cancer

The mean, median and distribution of utility scores for each health state at ‘12 months’ and ‘lifetime’ duration are shown in Table 1 and Figs. 2 and 3. Mean utility scores were higher for ‘screened, cytology normal’, ‘HPV vaccination’ and ‘HPV positive, endoscopy normal’ (ranged from 0.87 to 0.92) than for ‘early stage invasive throat cancer’ (ranged from 0.75 to 0.79). Lower mean utility scores were observed for ‘early stage invasive throat cancer’ with 0.75 at 12 months and 0.79 for lifetime duration. In addition, the interquartile range for ‘screened, cytology normal’ was ‘0.96 to 1.00’ when anchored to 12-month duration for ‘early stage invasive throat cancer’ and ‘0.90 to 1.00’ when anchored to lifetime duration. This compared with, for ‘early stage invasive throat cancer’, ‘0.19 to 1.00’ for 12-months’ duration, indicating great heterogeneity in the evaluation of throat cancer compared to other health states.

The intraclass correlation coefficient between results which anchored to ‘12-months’ versus ‘lifetime duration’ for ‘early stage invasive throat cancer’ was 0.99 for most health states, except ‘early stage invasive throat cancer’ (ICC: 0.46) (Table 1). This indicates absolute agreement for most health state utility scores assessed using a ‘12-month’ and ‘lifetime’ duration.

The utility scores based on anchoring to the ‘12-month’ and ‘lifetime’ early stage throat cancer scores stratified by demographic characteristics are shown in Table 2. Younger participants (aged 18–40 years) had significantly higher utility scores in ‘screened, cytology normal’ and ‘HPV vaccination’ health states than older participants when anchored to both 12-month and lifetime early stage throat cancer (‘0.92–0.93’ vs ‘0.88–0.89’). However, the mean utility score was lower in the younger age group (0.85) than the older age group (0.89) when anchored to lifetime duration in ‘early stage invasive throat cancer’.

**Table 2:** Age, sex and residential location comparisons of standard gamble utility scores using the ‘12 month’ and ‘lifetime’ duration early stage throat cancer (*n* = 1011)

		Mean (95%CI)	Mean (95% CI)	
	**Health states**	**Age groups (years)**		***P*-value**
		**18–40** (*n* = 539)	**> 40** (*n* = 467)	
**12 month-early stage throat cancer**	**S1**	0.93 (0.90–0.95)	0.89 (0.86–0.90)	0.0319
	**S2**	0.94 (0.92–0.96)	0.88 (0.85–0.91)	0.0022
	**S3**	0.91 (0.89–0.93)	0.92 (0.89–0.94)	0.6276
	**S4**	0.88 (0.85–0.91)	0.87 (0.84–0.91)	0.8666
	**S5**	0.85 (0.82–0.88)	0.89 (0.86–0.92)	0.0682
	**S6**	0.74 (0.70–0.78)	0.76 (0.72–0.80)	0.3715
**Lifetime duration-early stage throat cancer**	**S1**	0.92 (0.91–0.94)	0.89 (0.86–0.90)	0.0220
	**S2**	0.94 (0.92–0.96)	0.88 (0.85–0.91)	0.0012
	**S3**	0.91 (0.89–0.93)	0.92 (0.89–0.94)	0.4072
	**S4**	0.88 (0.86–0.91)	0.87 (0.84–0.90)	0.1565
	**S5**	0.85 (0.82–0.87)	0.89 (0.88–0.92)	0.0456
	**S6**	0.78 (0.74–0.81)	0.81 (0.77–0.84)	0.1970
	**Health states**	**Sex**		***P*-value**
		**Male** (*n* = 340)	**Female** (*n* = 671)	
**12 month-early stage throat cancer**	**S1**	0.92 (0.89–0.95)	0.90 (0.88–0.93)	0.9539
	**S2**	0.89 (0.86–0.92)	0.93 (0.91–0.95)	0.1330
	**S3**	0.90 (0.87–0.94)	0.91 (0.89–0.94)	0.5348
	**S4**	0.86 (0.82–0.90)	0.89 (0.86–0.91)	0.4739
	**S5**	0.86 (0.82–0.90)	0.87 (0.85–0.90)	0.6064
	**S6**	0.70 (0.65–0.73)	0.77 (0.74–0.80)	0.0262
**Lifetime duration-early stage**	**S1**	0.91 (0.89–0.94)	0.90 (0.88–0.92)	0.8810
	**S2**	0.89 (0.86–0.92)	0.92 (0.90–0.94)	0.2170
	**S3**	0.91 (0.88–0.94)	0.92 (0.89–0.94)	0.5278
	**S4**	0.86 (0.82–0.90)	0.89 (0.87–0.91)	0.6202
	**S5**	0.86 (0.82–0.90)	0.87 (0.85–0.90)	0.4975
	**S6**	0.76 (0.72–0.81)	0.81 (0.77–0.84)	0.1204
	**Health states**	**Location**		***P*-value**
		**Metropolitan** (*n* = 376)	**Regional** (*n* = 633)	
**12 month-early stage throat cancer**	**S1**	0.91 (0.89–0.93)	0.91 (0.88–0.94)	0.9706
	**S2**	0.91 (0.89–0.93)	0.92 (0.90–0.95)	0.5519
	**S3**	0.92 (0.90–0.94)	0.90 (0.87–0.93)	0.4017
	**S4**	0.89 (0.87–0.92)	0.86 (0.82–0.89)	0.0659
	**S5**	0.89 (0.87–0.92)	0.82 (0.79–0.86)	0.0005
	**S6**	0.82 (0.79–0.85)	0.63 (0.58–0.68)	< 0.0001
**Lifetime duration-early stage**	**S1**	0.91 (0.88–0.93)	0.91 (0.88–0.93)	0.8955
	**S2**	0.91 (0.89–0.93)	0.92 (0.89–0.95)	0.6136
	**S3**	0.92 (0.90–0.94)	0.90 (0.87–0.93)	0.2756
	**S4**	0.89 (0.87–0.92)	0.86 (0.82–0.89)	0.0555
	**S5**	0.89 (0.87–0.92)	0.82 (0.79–0.86)	0.0005
	**S6**	0.87 (0.85–0.90)	0.66 (0.61–0.70)	< 0.0001

Notes: *P*-value: Wilcoxon rank sum test. S1: screened, cytology normal. S2: HPV vaccination. S3: screened; HPV positive, endoscopy normal. S4: oral warts. S5: high grade cytology with OIN II-III. S6: early stage invasive throat cancer

There were no significant differences in mean utility scores between males and females for most health states, either when anchored to 12-months or lifetime duration, except ‘early stage invasive throat cancer’ at 12 months duration (males had a lower mean score). Metropolitan-dwelling participants had significantly higher utility scores for ‘oral intraepithelial neoplasia’ and ‘early stage invasive throat cancer’ than those residing in non-metropolitan locations for both the 12-month and lifetime duration anchors of early stage throat cancer.

## Discussion

This study is the first to work in partnership with Indigenous stakeholders to develop, pilot test and modify health state descriptions in relation to oral HPV infection and OPSCC from the perspectives of Indigenous Australians, and then evaluate utilities for these health states in a large convenience sample of the same. This study would not have been possible without the establishment of genuine research partnerships with South Australian Indigenous communities. Our findings demonstrated that the more severe the health state, the lower the utility score, no matter the length of duration of the anchor state. The negative impact of throat cancer on quality of life was much greater for people living in regional areas compared to those in metropolitan areas. Our findings broadly reflect those reported by Simonella and colleagues for health states for cervical screening and abnormalities among a general population of women [29], and from Noel and colleagues among patients aged 50 years and above with head and neck cancer [32]. The utility score (0.75) for ‘early stage invasive throat cancer’ also closely matches the estimate reported by Rogers and colleagues for oral cancer and OPSCC [33].

It is interesting that Indigenous women in our study had higher utility scores for the ‘early stage invasive throat cancer’ health state at both 12-months and lifetime duration than Indigenous men. This finding is consistent with research by Banham and colleagues on Indigenous South Australians’ cancer treatment [34], who reported that Indigenous females received more treatment, such as surgeries, systemic therapy and radiotherapy than males, on average. The authors concluded that this was consistent with health-related gender differences observed in the wider Australian population [32].

The lower utility scores observed for the health states ‘oral intraepithelial neoplasia’ and ‘early stage invasive throat cancer’ for non-metropolitan participants may reflect socioeconomic status, with those not living in a city having lower income, perhaps struggling to meet the financial and associated costs related to treatment, or experiencing greater discrimination in the hospital setting. It potentially also reflects the much greater distance that people living outside metropolitan areas need to travel to access tertiary hospital treatment in South Australia, which is predominantly located in the capital, Adelaide. Evidence from Kelly and colleagues [35], who developed mapping tools with Aboriginal patients to better quantify Aboriginal experiences in hospital journeys, supports this.

There are three main strengths of the study. The first is the extensive development, pilot testing and refinement of the health states conducted through an Indigenous Reference Group. This is considered essential in any stage of developing tools for use with and by Indigenous Australians. The second is that the health states were then used in a large convenience sample of Indigenous South Australians. The study had extremely good Indigenous community buy-in, with some participants going to considerable length to contact the research team to enquire what they needed to do to be involved. The third strength is the use of a two-stage standard gamble approach, yielding helpful data for cost effective and health economic analysis. Limitations include participants being based in South Australia and recruited through ACCHOs only, meaning the findings may not be generalisable to the many other culturally and linguistically diverse Indigenous groups elsewhere in Australia. The findings, for the same reason, may also not be generalisable to other Indigenous groups in the world. In addition, our data had a bimodal distribution. However, the percentage of the larger mode (the major modal) was around 80%, ranging from 78 to 90%, (see Figs. 2 and 3), which conformed to a real ‘central tendency’ of our data.

Although there is no a standard screening to prevent OPSCC at a population level, each time a person receives dental care early signs of oral cavity cancer and OPSCC are checked. HPV status is a strong prognostic factor for survival rate. The prognosis for the HPV-positive OPSCC is overall better than HPV-negative OPSCC according to the American Joint Committee on Cancer (AJCC) 8th Edition guidelines [36]. Further work is needed to assess the difference in utility between HPV-positive and negative oropharyngeal cancer; between Indigenous and non-Indigenous Australians, and among population groups at an international level.

## Conclusion

Six health states representing HPV infection and vaccination, oral screening and screen-detected abnormalities, and OPSCC from the perspective of Indigenous Australians were successfully developed using culturally respectful processes. The reduction in quality of life was perceived to be greater with increasing severity of health states. There were differences observed by geographic location, with negative throat cancer-related quality of life being much higher among regional-dwelling participants. Younger participants had higher quality of life scores for HPV-related disease prevention (vaccination and screening) and lower quality of life scores for throat cancer compared with older participants. Our findings are an important contribution to cost-utility and disease prevention strategies that seek to inform policies around reducing HPV infection and OPSCC among all Australians. The information could be used to directly calculate quality-adjusted life years and to, in turn, be translated into health policy regarding Indigenous patient journeys with primary and secondary prevention for HPV-related OPSCC.

## Supplementary Information

**Additional file 1.** Health state vignettes.

## Data Availability

The datasets generated and/or analyzed during the current study are not publicly available due to privacy issues of the participants. Data are available from the corresponding author on reasonable request.

## Abbreviations

HPV: Human papillomaviruses
ICC: Intraclass correlation coefficient
IRG: Indigenous Reference Group
OIN: Oral intraepithelial neoplasia
OPSCC: Oropharyngeal squamous cell carcinoma
