# Supplementary material for: Population-based utility scores for HPV infection and oropharyngeal squamous cell carcinoma among Indigenous Australians
Source: BMC Public Health. 2021 Jul 26;21:1455. doi: 10.1186/s12889-021-11496-z (PMC8314643; doi:10.1186/s12889-021-11496-z)

**Appendix A: Health state vignettes**

**Health state: Screened; cytology normal (S1)**


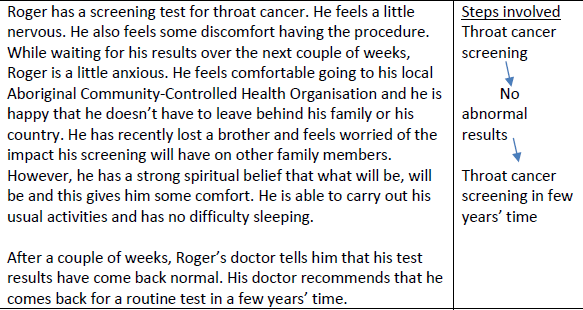


**Health state: HPV vaccination (S2)**


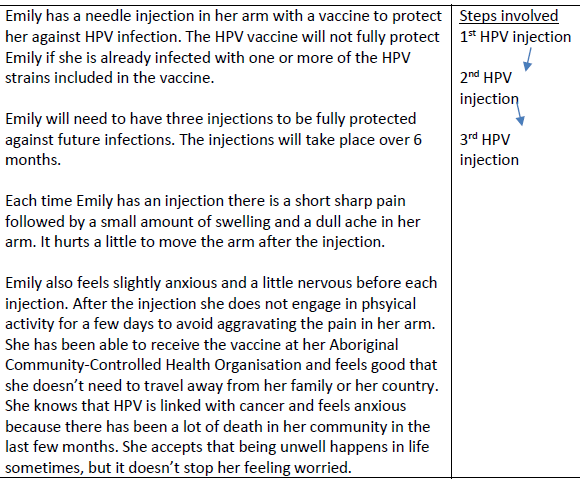


**Health state: HPV positive, endoscopy normal (S3)**


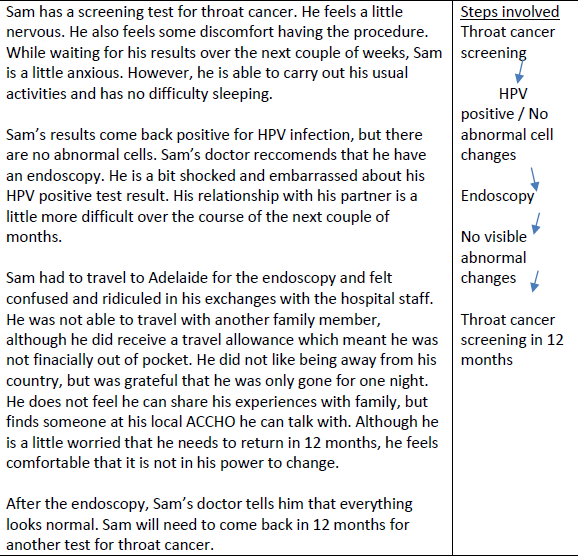


**Health state: Oral warts (S4)**


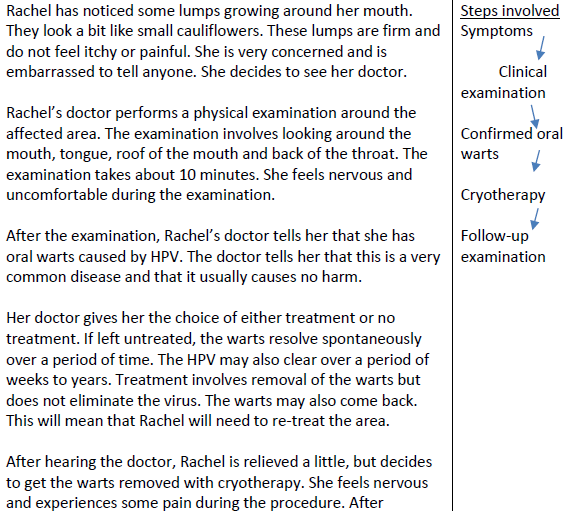

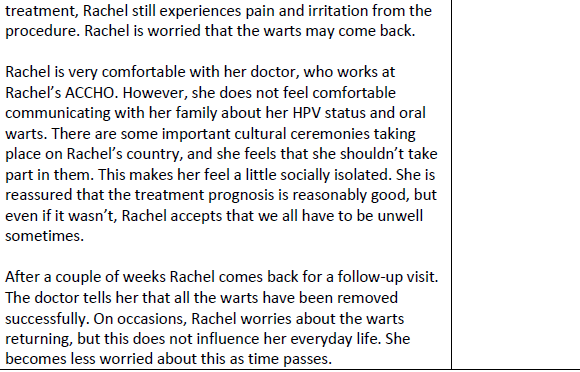


**Health state: High grade cytology with histologically-confirmed Grade II/III oral intraepithelial neoplasia (S5)**


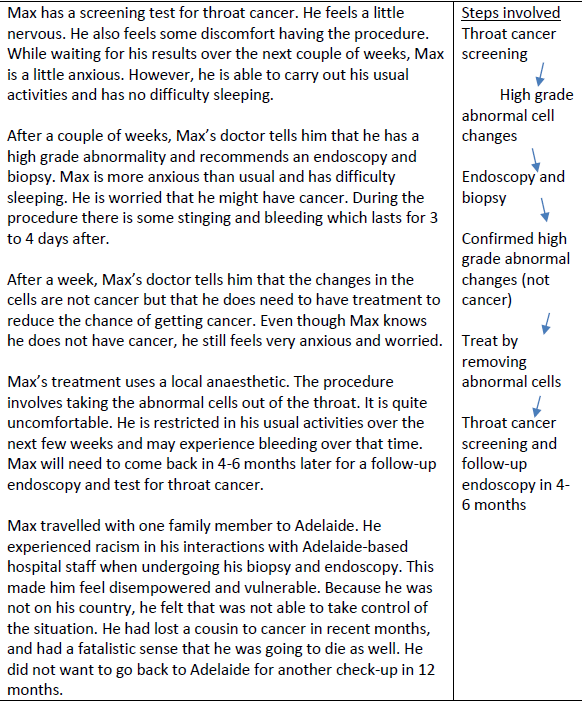


**Health state: Early stage invasive throat cancer (S6)**


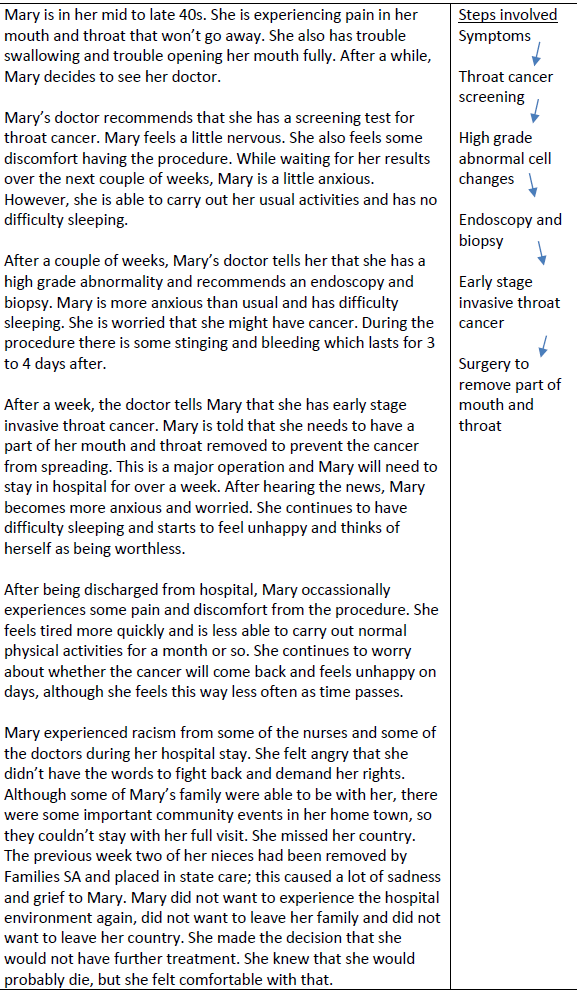

Supplement: Supplementary file 1 — Additional file 1. Health state vignettes. [file 12889_2021_11496_MOESM1_ESM.docx]
